# Low Field Optimization of a Non-Contacting High-Sensitivity GMR-Based DC/AC Current Sensor

**DOI:** 10.3390/s21072564

**Published:** 2021-04-06

**Authors:** Cristian Mușuroi, Mihai Oproiu, Marius Volmer, Jenica Neamtu, Marioara Avram, Elena Helerea

**Affiliations:** 1 Department of Electrical Engineering and Applied Physics, Transilvania University of Brasov, Blvd. Eroilor 29, 500036 Brasov, Romania; cristian.musuroi@unitbv.ro (C.M.); mihai.oproiu@unitbv.ro (M.O.); helerea@unitbv.ro (E.H.); 2 National Institute for Research and Development in Electrical Engineering, Splaiul Unirii 313, 030138 Bucharest, Romania; jenica.neamtu@gmail.com; 3 National Institute for Research and Development in Microtechnologies, Str. Erou Iancu Nicolae 32B, 72996 Bucharest, Romania; marioara.avram@imt.ro

**Keywords:** current sensors, GMR effect, magnetoresistive sensors, bias magnetic field, Biot-Savart law, magnetic nanoparticles

## Abstract

Many applications require galvanic isolation between the circuit where the current is flowing and the measurement device. While for AC, the current transformer is the method of choice, in DC and, especially for low currents, other sensing methods must be used. This paper aims to provide a practical method of improving the sensitivity and linearity of a giant magnetoresistance (GMR)-based current sensor by adapting a set of design rules and methods easy to be implemented. Our approach utilizes a multi-trace current trace and a double differential GMR based detection system. This essentially constitutes a planar coil which would effectively increase the usable magnetic field detected by the GMR sensor. An analytical model is developed for calculating the magnetic field generated by the current in the GMR sensing area which showed a significant increase in sensitivity up to 13 times compared with a single biased sensor. The experimental setup can measure both DC and AC currents between 2–300 mA, with a sensitivity between 15.62 to 23.19 mV/mA, for biasing fields between 4 to 8 Oe with a detection limit of 100 μA in DC and 100 to 300 μA in AC from 10 Hz to 50 kHz. Because of the double differential setup, the detection system has a high immunity to external magnetic fields and a temperature drift of the offset of about −2.59 × 10^−4^ A/°C. Finally, this setup was adapted for detection of magnetic nanoparticles (MNPs) which can be used to label biomolecules in lab-on-a-chip applications and preliminary results are reported.

## 1. Introduction

Current measurement is essential in modern electrical systems. Different current sensing methods have been developed and adapted for specific needs. Resistive-based current-sensing techniques, although acceptable to use in some applications, have a number of drawbacks such as low accuracy, power loss, low bandwidth, no galvanic isolation, and noise [1]. In contrast, electromagnetic-based current sensing techniques, mitigate most of these drawbacks, but present some specific challenges regarding their operation or application versatility.

However, many applications require galvanic isolation between the circuit where the current is flowing and the measurement device. Because of that, magnetic sensors are widely used in current measurements because they are non-intrusive and provide galvanic isolation. A good example of non-contact current detection, that can inspire many other applications, is presented in [2] where the ion beam equivalent current inside a particle accelerator, is measured through the magnetic field it creates. In this way, equivalent currents down to 100 μA can be measured. Typical current sensors include the AC/DC current transformers [2,3,4], fluxgate magnetometers [5,6], Hall effect sensors [3,7], anisotropic magnetoresistive (AMR) [3,8], giant magnetoresistance (GMR) [2,3,9,10] and tunnelling magneto-resistance (TMR) sensors [2,11].

For modern applications, some essential features for current sensors can be noted as necessary: enhanced accuracy and sensitivity, linear response, versatile DC/AC measurement, low thermal drift, immunity to interferences, integrated circuit (IC) packaging, low power consumption and low cost. 

(Micro)fluxgate sensors [3,6] are an established solution for DC/AC currents detection since they offer good accuracy and stability. A fluxgate sensor [3] is typically comprised of high permeability magnetic cores around which coil windings are wrapped: a fluxgate coil (driven by a square wave current), compensation coil and pick-up coil which are used to determine the magnetization state of the core and, hence, the current to be measured. The necessary setup for managing the sensor’s functionality, and conditioning the acquired signal is quite complex. While the classical setup is using discrete parts like magnetic core and coils wrapped on it [3], the modern solutions are using integrated microfluxgate sensors, which can be placed directly around the conductor [6]. 

By contrast, Hall microsensors can simplify the measurement setup and are compatible with IC technology [12] which means that both the sensing part and the electronics required for signal conditioning can be microfabricated on the same chip. Hall sensors do not saturate and can reach a sensitivity down to 10^−6^ T [13]. However, these sensors do require a closed magnetic core with a small air gap where the Hall sensor is placed [3,7]. The core surrounds the conductor through which the current is flowing. It must be mentioned that magnetic core becomes a source of non-linearity for the response characteristic due to hysteretic effects. Also, serious DC offset of the sensor’s output can be caused by the remanence of the magnetic core. Some Hall current meters have an AC demagnetization circuit to overcome this issue [3]. There are manufacturers that integrate on the same chip the current trace, the Hall sensor and the conditioning circuits giving a compact solution, usually suitable for currents larger than 5 A.

On the other hand, magnetoresistive sensors (MR) made of magnetic thin films can exhibit much higher sensitivities, being able to detect magnetic fields in the 10^−9^–10^−2^ T domain [13]. This can lead to simplification of the current measurement chain, the sensor being sensitive to in-plane magnetic fields, extending the current measurement range for values lower than 1 mA, allowing the possibility for developing special applications with reduced power consumption and price [8,9,11,14]. Also, it should be mentioned that for MR sensors the response characteristics like sensitivity, linearity, saturation field, noise, etc., are strongly affected by the magnetic properties of the utilized materials, the structure of their underlying multilayered and the layout of the microfabricated sensor. A short review of MR effects, underlying the physical origin of AMR, GMR and TMR, and the specific field behavior was presented in [10].

The GMR effect occurs in multilayered magnetic structures of the type FM/NM/FM coupled by exchange interaction; here FM denotes magnetic layers like Ni_80_Fe_20_, Co, CoFeB, etc., and NM denotes a nanometer thick conductive nonmagnetic layer as Cu, Ag. If the NM is a dielectric of the type Al_2_O_3_ or MgO_2_ we talk about a TMR structure [10,11]. In GMR effect the resistance changes according to the angle between the directions of the magnetization of adjacent layers. When the layers are magnetized in parallel, the resistance is at a minimum value, *R_p_*. When the magnetizations of the adjacent magnetic layers are antiparallel to each other, the resistance is at a maximum value, named *R_ap_*. The magnitude of the GMR effect is expressed by Equation (1) [15], and is typically between 5–15%:

(1) $$GMR=\frac{R_{ap}-R_{p}}{R_{ap}}100\;\;\left[\%\right],$$

Usually, one of the ferromagnetic layers is pinned by an anti-ferromagnetic (AFM) layer of FeMn or IrMn while the second FM layer has the magnetization free to rotate under the action of an external magnetic field. Such that, a GMR structure is of the type substrate/buffer layer (Ta)/FM(free layer)/NM/FM(pinned layer)/AFM/Cap layer [10].

Compared with AMR based sensors, the GMR sensors offer higher field sensitivity, wide frequency range, and do not require an internal coil (for example, of type KMZ51) or external controlled magnetic field to be used to reset the magnetization to the initial orientation [8]. However, most of the GMR sensors have a nonlinear behaviour around zero field and the output is unipolar which limits the application for bipolar and AC fields [9,10]. Until now, several methods of improving the response of a GMR sensor have been used. For example, using a bias field parallel to the sensitive axis can shift the operating point of the sensor in the linear region, thus reducing hysteresis behaviour and allowing detection of bipolar fields. In [10], we mentioned that this field can be created with a permanent magnet or a coil system with DC, AC, or short pulse currents which can have open or closed-loop control. 

Several studies have presented the use of GMR sensors as current sensors, most detailing different methods to improve their performance and versatility. Some of the implemented methods are: magnetic shielding—for reduced susceptibility to interference magnetic fields [16], hysteresis modelling compensation [17], open loop operation [2,3,10], closed loop operation [18]—to reduce hysteresis and temperature dependency, low frequency capture—to increase the range of the GMR current sensor to ±800 A [19], negative feedback introduced by the Helmholtz coil—to increase the range of the sensor up to 5 times [9], or damping coil—to further increase the range by 5.23 times [20]. In [10], we demonstrated a novel method of improving the overall accuracy, thermal stability, power consumption and immunity to interference magnetic fields involving a double differential setup, adjustable permanent magnetic biasing and antiphase-operating GMR based sensors on top of a U-shaped current trace. As a general remark from the above enumeration, many current sensors are using: (i) a discrete solution, where the magnetic sensors, the current trace, coils and the corresponding electronics are implemented in a suitable setup to detect the current [2,3,8,9,10,11] or (ii) a compact solution as an integrated circuit where can be found inside the chip the current trace, the sensors [13] and the conditioning circuit [14]. 

In this paper, which continues the study presented in [10], an extremely sensitive GMR current sensor is designed and implemented, able to detect both DC and AC currents from 2 to 300 mA with a setup sensitivity between 15.62 to 23.19 mV/mA. The detection limit is 100 μA in DC and 100 to 300 μA in AC from 10 Hz to 50 kHz. We found this limitation to be due mainly to the signal processing chain and not due to the GMR sensors which have an operating frequency range from DC to 1 MHz. The basic approach is to use a multi-turn planar coil and a double differential GMR based detection system. To study the influence of the biasing field on the current sensor sensitivity and linearity and to fine balance the GMR sensors, a Helmholtz coil is used. 

Finally, further applications of this measurement system will be discussed. We proved that this system is able to detect very low amounts of magnetic field generated by magnetic nanoparticles placed above the sensor surface.

## 2. Materials and Methods

### 2.1. Principle of Operation

**Figure 1:**
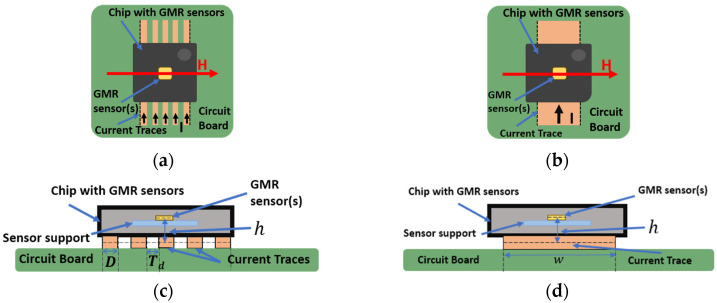
Working principle of the non-contacting current measurement setup utilizing current traces and a GMR based chip: (**a**) Multi-trace plane section; (**b**) Single trace plane section; (**c**) Multi-trace cross section; (**d**) Single trace cross section.

**Figure 2:**
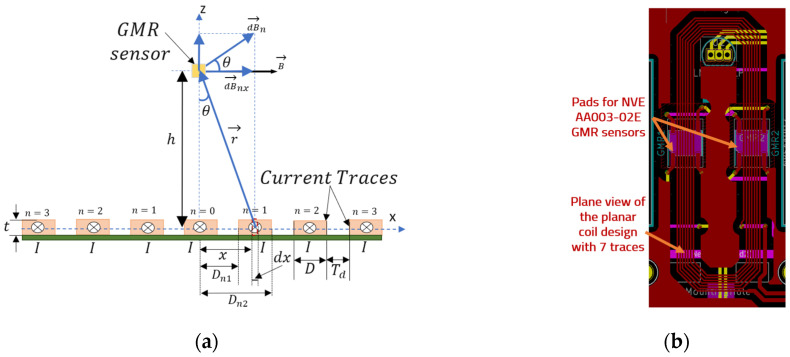
(**a**) Illustration of the geometry and parameters used in the analytical model to compute the magnetic field present in the sensor area. Note that the model takes into account that there is an odd number of traces that generate the magnetic field and the central trace is denoted as *n* = 0; (**b**) plane view of the planar coil with seven traces as designed for the actual implementation. $\vec{B}$ is the resulting field in the central position (the middle trace is centered just below the sensitive area of the sensor).

The proposed setup relies on a practical method of significantly increasing the sensitivity and accuracy of a non-contacting current sensor by an appropriate design of the circuit which produces the magnetic field from the current that is intended to be measured. In order to validate this concept, GMR sensors were used. The novelty of the approach consists in utilizing multiple current traces, in a double differential system, implemented in a custom printed circuit board. In the current measurement setup, the GMR sensors act as a magnetometer, thus, if a current, *I*, passes through a wire, the magnetic field, *B*, will produce a change of the output voltage on the nearby GMR sensor. The working principle of the setup can be seen in Figure 1a,c. As a particular case, we can note the single trace variant, illustrated in Figure 1b,d and described in detailed in [10]. As shown in [10], the supposition for this setup is that the low current measurement capabilities can be significantly improved by utilizing a narrow trace. Moreover, by having multiple traces through which the same current to be detected passes, the system will become a planar coil (Figure 2b). The current, *I,* from the conducting traces (denoted as “Current traces”), generates a magnetic field, whose component, *B_x_*, will be detected by the GMR sensor (note that this setup can be adapted to work with other types of sensors, for example, planar Hall effect sensors).

In order to estimate *B_x_*, we derived an analytical model based on Biot-Savart law, which assumes that the sensor is centered above the multiple trace at distance *h* (Figure 2a).

Thus, by assuming a long conducting trace, as seen in the design from Figure 2b, the elementary field produced by the current *I*, can be expressed, using the Biot-Savart law, by Equations (2) and (3):

(2) $$dB_{n}=\mu_{0}\frac{dI}{2\pi r}=\mu_{0}\frac{Idx}{D}\cdot \frac{1}{2\pi \sqrt{h^{2}+x^{2}}}\;;dI=\frac{I}{D}dx,$$

(3) $$dB_{nx}=dB_{n}\cdot \cos\theta =\mu_{0}\frac{Idx}{2\pi D}\cdot \frac{1}{\sqrt{h^{2}+x^{2}}}\cdot \;\frac{h}{\sqrt{h^{2}+x^{2}}}\;,$$

where *µ*_0_ = 4*π* × 10^−7^ H/m is the vacuum magnetic permeability, *D* is the trace width, *t* is the trace thickness (not used in the equation, *T*_d_ is the distance between the traces, *h* is the height on which the sensing element is placed above the trace, and *θ* is the angle shown in Figure 2a used to estimate the *B_x_* component of the magnetic field.

By assuming a uniform linear current density, *I*/*D*, and integrating Equation (3) from *D_n_*_1_ to *D_n_*_2_ we find the *x* component of field generated by a trace *n* = 1, 2, 3,…, in the sensor area:

(4) $$B_{nx}=\frac{\mu_{0}I}{2\pi D}\left[arctan\left(\frac{D_{n2}}{h}\right)-arctan\left(\frac{D_{n1}}{h}\right)\right]\;\;\left[T\right]$$

Note that for the central trace (*n* = 0), B_0*x*_ was found in [10] to be:

(5) $$B_{0x}=\;\frac{\mu_{0}I}{\pi D}\cdot arctan\left(\frac{D}{2h}\right)\;\;\left[T\right]$$

Taking into account the problem symmetry, the z component of the field in the sensor will be canceled by the fields from the left and right-side stripes, i.e., B_nz_ = 0. Such that, the total field generated in the sensor area can be expressed as:

(6) $$B=B_{0}+2B_{1}+2B_{2}+\ldots +2B_{n}\;\left[T\right]$$

For example, if we consider the situation illustrated in Figure 2a, for *n* = 3 it means that there will be seven linear stripes beneath the sensor that will produce the magnetic field (Figure 2b). In fact, this is the actual planar coil used in our practical implementation, where *h* = 0.8 mm, *D* = 0.22 mm and *T*_d_ = 0.19 mm. For these parameters we find that (note that the current *I* is expressed in [A]):

(7) $$B=10.3784\cdot I\cdot 10^{-4}\left[T\right]=10.3784\cdot I\;\left[G\right]$$

Note that even though the results from Equations (4)–(6) are expressed in Tesla, they can be transformed into Gauss (which is equivalent to Oe in air) by multiplying by 10^4^. 

**Figure 3:**
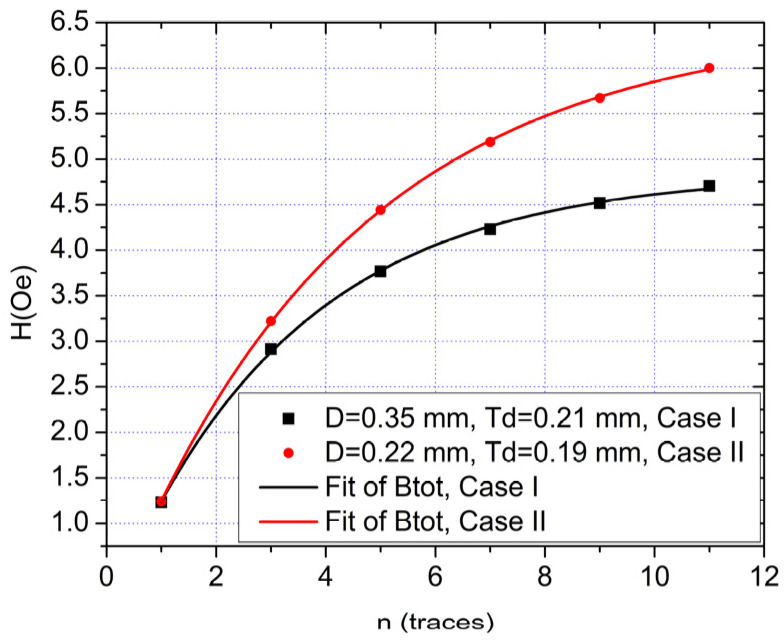
Total magnetic field induction in the GMR sensor area (for I = 0.5 A) dependency on the number of current traces. The asymptotic tendency of Btot for the case where *D* = 0.22 mm is 6.485 Oe (Case I) while for *D* = 0.35 mm is 4.849 Oe (Case II). In both cases, *h* = 0.8 mm. Note that the field multiplying effect is clearly visible. Graphs were made using data obtained with the analytical method.

For ease of use as well as testing various scenarios, meaning different values for *n*, *h*, *D* and *T_D_*, this analytical method was implemented in a LabVIEW application. Note that for only one trace (*n* = 0), the result is detailed in [10]. An analysis was performed with this method for two different trace structures (defined by the D and *T*_d_ parameters, Figure 2a). The field dependency between the two cases, for different number of traces, as obtained with the analytical algorithm can be seen in Figure 3. An example of the detailed calculus for one of the structures (Case II, Figure 3) is shown in Appendix A, while the parameters involved are shown in Table A1 and Equations (A1) and (A2), the example calculation being shown in Equations (A3)–(A13).

From Figure 3, we can note that the magnetic field in the sensor area is increased significantly from the case with just one trace (the results were obtained with a 0.5A current passing through each trace). The best balance between the obtained field and practicality of implementation was found to be at the five and seven traces structure as there is an asymptotic dependency. Thus, a prototype PCB for current sensing with the specifications from Case II (for higher magnetic field output), Figure 3, was be developed. The values of *D* and *t*_D_ were chosen such that to get the maximum field in sensors region and to allow a higher current to pass through the planar coil. The values of *D* and *t*_D_ used to plot Figure 3 show that: (i) the developed analytical method can take into account different trace widths and spacing to estimate the produced magnetic field and (ii) a large number of traces with a lower value of *D* give higher value for *B* in the sensor region. The lower value for *t*_D_ was 0.19 mm due to technological limitations. Because our work was focused on measuring low currents down to 1 mA, with a high dynamic range, up to 300 mA, and because of the physical distance between the sensor pads, we set *D* = 0.21 mm. 

We can denote that other factors also play a major role in the sensitivity of the sensors (such as biasing of the sensors, differential measurement setup, amplifying the output etc.) [10]. Thus, we can expect much better results with an optimized designed of the setup and appropriate biasing of the GMR sensors. From the results obtained with the analytical algorithm, we estimate that compared with a single biased NVE AA003-02 GMR sensor (supplied with 1 mA of current, average sensitivity *S* around 13.75 mV/Oe), by using multiple traces (for example, 7) on an optimized double differential design as in [10], we can expect a sensitivity *S* increase (for a 500mA measured current) from *S* = 0.0341 mV/mA (single trace) to *S* = 0.244 mV/mA (five traces, Case I) or *S* = 0.285 mV/mA (seven traces, Case II), Figure 3. 

### 2.2. Characterization of the GMR Sensor

**Figure 4:**
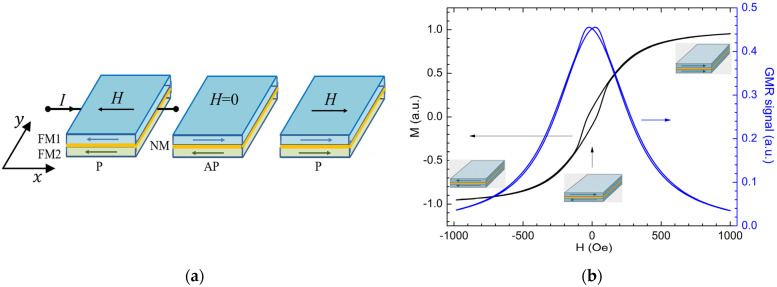
Micromagnetic simulation of a GMR element: (**a**) Schematic illustration of a GMR structure (two ferromagnetic layers FM1, FM2 and nonmagnetic layer, NM) with three distinct states depending on the parallel (P) or antiparallel (AP) alignment of layers magnetization; (**b**) Typical field dependence of the structure magnetization along the Ox axis, M, obtained by micromagnetic simulations and the calculated GMR effect when *H_appl_* is directed over Ox axis.

Commonly, GMR sensors are made from multilayered structures of the type AFM (antiferromagnetic layer)/PL(pinned magnetic layer)/NM(non-magnetic spacer layer)/ FL(magnetic free layer). The free layer is the sensing layer, as the magnetization can rotate upon an applied magnetic field). The difference in the relative magnetic moments between adjacent magnetic layers produces a change in the electric resistance. When the layers are magnetized in parallel, the resistance is at a minimum value, *R_p_*, which is the saturation resistance. When the magnetizations of the adjacent magnetic layers are antiparallel to each other, the resistance is at a maximum value, named *R_ap_* (note Figure 4a). The electric resistance dependency from the angle *θ,* between the magnetization of adjacent magnetic layers [21]:

(8) $$R=R_{p}+\frac{\Delta R_{GMR}}{2}\left[1-cos\theta \right],$$

where, Δ*R_GMR_* = *R*_p_ − *R*_ap,_ is the GMR effect amplitude. 

Thus, for antiparallel configuration (*θ* = 180°):

(9) $$cos\theta =-1\;\to R=R_{AP}=R_{High},$$

while, for parallel configuration (*θ* = 0°):

(10) $$cos\theta =1\;\to R=R_{P}=R_{Low}.$$

From Equations (8)–(10), we can express the rate of change in the resistance of a GMR sensor element (also called GMR ratio) by Equation (1). 

The AA003-02 sensor, which will be used in the experimental setup, contains two active GMR elements, and two magnetically shielded identical sensors used to complete a Wheatstone bridge; the GMR ratio for each sensor element is between 13% to 16% [22]. Thus, in 0 field the bridge is almost balanced providing an output voltage close to 0 [22], as will be showed from experimental results. When a field is applied, the bridge becomes unbalanced and the output voltage shows a sensitivity between 3–4.2 mV/(V × Oe) [10,22].

For a better understanding on the operation of the GMR sensor, by simulating this effect with the object oriented micromagnetic framework (OOMMF) [23], using a multi-domain approach, the layer orientation of a GMR structure in different operating points can be illustrated. The main parameters involved in configuring the simulation are: the simulated layer is 1000 × 500 × 10 nm^3^ and consists from Permalloy; the cell size is 5 × 5 × 5 nm^3^. The FL is antiferromagnetically coupled with the PL through the NM layer, the coupling field being 200 Oe, along the Ox axis. The magnetic field, *H_appl_*, is applied perpendicular to the easy axis of magnetization. The saturation magnetization *M*_s_ = 710 kA/m, the exchange constant *A* = 1.3 × 10^−11^ J/m, and the anisotropy constant *K*_U_ = 804 J/m^3^ along Ox axis, Figure 4. In [10] we described in detail the multi-domain micromagnetic approach (as well as the reasoning behind the parameters involved and how to extract simulation results) to simulate a GMR sensor structure using OOMMF.

**Figure 5:**
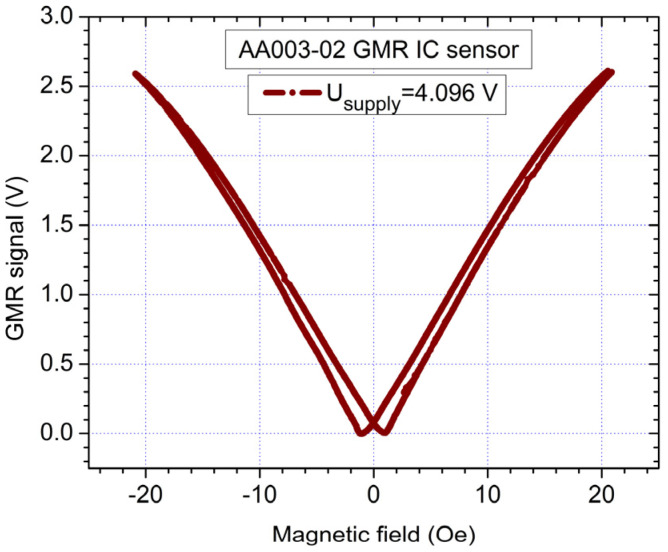
Typical measured magnetic field dependency for the AA003-02 GMR sensor. In this case, the sensor was supplied with 4.096 V; this voltage is generated by a very stable source which is part from the EI 1040 instrumentation amplifier.

In order to characterize the AA003-02 sensor (Figure 5), a magnetic field was applied by two round-shaped coils in a quasi-Helmholtz like configuration. From Figure 4 and Figure 5, we can observe that the results are in good qualitative agreement with Equations (8)–(10). We can remark that the sensor presents a nonlinear response around 0 field and low sensitivity around the coercive field. We noted that when supplying the sensors with a constant current (2 mA), instead of voltage, the sensitivity of the sensors can be increased [10]. Due to practical reasons, a constant supply voltage was chosen for the sensors. 

### 2.3. Experimental Setup and Mode of Operation

**Figure 6:**
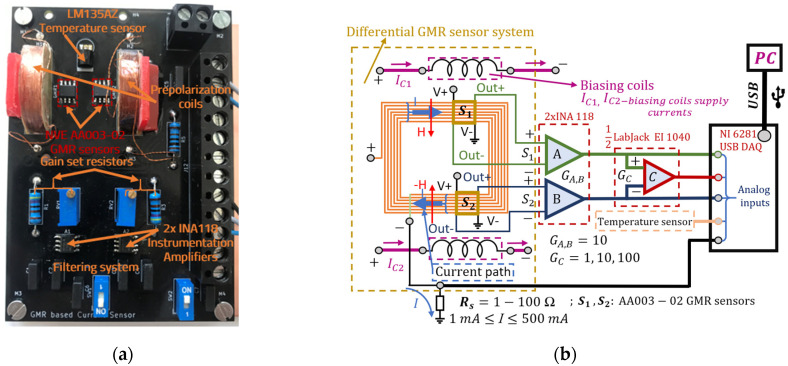
(**a**) GMR Testboard (custom PCB) optimized for low field detection (current measurement) using GMR sensors. The PCB is based on the 7 traces, Case II from Figure 3 and integrates the sensors, biasing coils, multi-turn planar coil, filtering system (capacitors for the INA 118 amplifiers power supply to filter any high frequency components and for the output of the amplifiers when measuring an AC signal to filter any DC component). (**b**) The functional block diagram of the current measurement system as well as the amplifier and data acquisition setup; notice the “U”-shaped structure of the circuit which produces the magnetic field which is applied to the sensors is integrated through the spiral trace which constitutes the planar coil.

**Figure 7:**
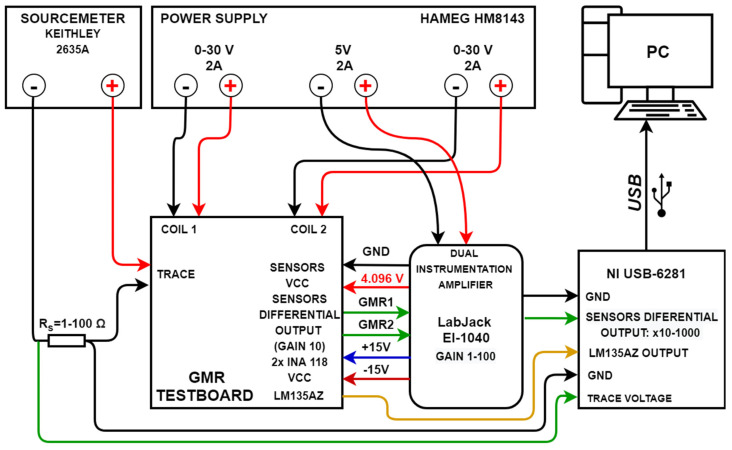
Subblock components of the entire setup. The GMR sensors, temperature sensor, as well as the INA118 amplifiers are supplied from the EI1040 instrumentation amplifier, which also allows a variable gain to be set as needed. On the GMR Testboard, the differential output from each sensor is amplified by a fixed gain of 10. The output voltage from the LM15AZ temperature sensor is sent directly to the DAQ board. Also note that each coil is supplied separately from the HM8143 (parallel configuration), as this allow calibration of the biasing field for each GMR sensor.

Based on the results from the analytical method, a GMR-based current measurement setup was developed. The PCB setup (named GMR Testboard) can be seen in Figure 6a. Figure 6b shows the functional block diagram of the current measurement system as well as the amplifier and data acquisition setup. Figure 7 details the individual subblock components of the entire setup. A HM8143 power supply (HAMEG, Frankfurt, Germany) is used for powering most components, while a 2635A Sourcemeter (Keithley, Solon, OH, USA) was used to generate the various measured trace currents. In terms of design rules for the current traces, a single trace in a U shaped spiral pattern (7 traces) passes through the sensors. Also, in order to amplify the useful magnetic field, the characteristics are those from Case II (note Figure 3). Due to the thickness of the trace, the setup is able to operate with currents up to 0.5 A, however, for testing we focused on values up to 150 mA, as higher values can easily be detected. The GMR sensors, from an output point of view, operate in antiphase, in a similar differential setup as the one detailed in [10], which provides some benefits: high sensitivity, immunity to any external homogenous magnetic field affecting both sensors equally or from magnetic fields lower than 25 Oe perpendicular to the axis of sensitivity. However, for non-homogenous magnetic fields in the vicinity of the sensors, electromagnetic shielding must still be applied. 

The output voltage from the sensors is amplified by two (one for each sensor) INA118 [24] instrumentation amplifiers integrated on the board. The real gain for each amplifier was set to 10 and the results were offset corrected. Furthermore, the resulting signals are further amplified by a LabJack EI1040 amplifier [25] to obtain the differential output from the two sensors which is set to a gain of 10 for low currents measurement, or 1 for higher currents measurement, resulting in a 10, 100 or 1000 total gain. The gain for the EI1040 instrumentation amplifier can be set manually or through the NI6281 USB, which represents the data acquisition board [26]. The implementation allows measurement of both AC and DC currents with the ability to bypass the included filtering system for the amplifier output. An LM135AZ temperature sensor (STMicroelectronics, Geneva, Switzerland) was also integrated in the sensor area. It should be noted that the supply lines for the GMR and temperature sensor are orientated perpendicularly compared with the current trace (any produced magnetic field lower than 20 Oe (Figure 4) is not detected by the GMR sensors) as to eliminate any additional magnetic field. 

The biasing coils are placed in a simmetrical quasi Helmholtz like configuration because of the requirement for ensuring an adequate distance between each GMR sensor (such as the traces beneath each sensor to not influence the output of the other sensor). The magnetic field strength in the sensor area (*H_bias_*_)_ was precisely determined during the PCB assembly stage (before sensor placement) using a 475 DSP Gaussmeter (Lake Shore Cryotronics, Westerville, OH, USA).

More studies can be done to find out the minimum possible distance between the GMR sensors to ensure proper results. This effect could have been mitigated by using larger biasing coils but that would have contributed to the costs and power consumption of the system. As a prepolarization field is necessary for linearizing the GMR sensors output, the biasing for the sensors was set to 4–8 Oe. The relationship between the coils supply current and the bias magnetic field present in the sensor area in air is (the current *I_C_* is expressed in mA):

(11) $$H_{bias}=I_{C}\cdot 0.1391\;\left[\mathrm{Oe}\right]$$

where *H_bias_* is the bias magnetic field produced by the coil in the sensor area and *I_C_* is the coil 1 and coil 2 (Figure 6b) supply current (e.g. 57.55 mA for 8 Oe bias). Note that the resistance of each biasing coil is 38 Ω.

Furthermore, like shown in [10], if we take into account the thermal influence for the sensors response, by considering that the system is thermally balanced and the same type of sensors are used, the total output voltage of the sensors from the differential system can be expressed with:

(12) $$\Delta U=(K_{S1}+K_{S2})\cdot H_{I}=(K_{S1}+K_{S2})\cdot C\cdot I=S\cdot I$$

where $K_{S1}$ and $K_{S2}$ are the sensitivities of each sensor, *S* (mV/mA) is the sensitivity of the differential measurement system, $H_{I}=C\cdot I$ (*C* is a constant) is the magnetic field strength created by the current passing through the trace.

## 3. Results and Discussion 

### 3.1. Experimental Results for the Current Measurement Setup

**Figure 8:**
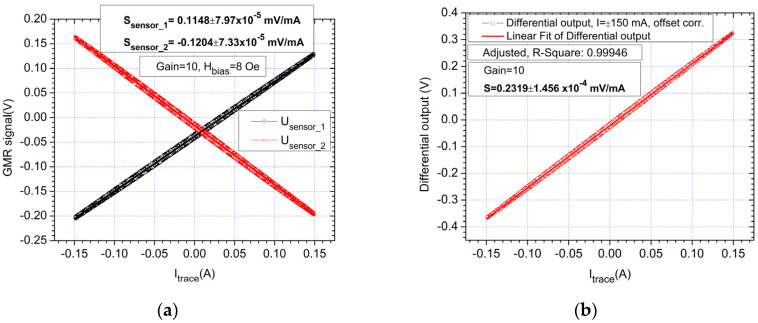
Response of the system for a ±150 mA, DC current, *H*_bias_ was set to 8 Oe: (**a**) individual sensors response; (**b**) differential output.

The results presented in this section are a summary and focus on demonstrating the low currents sensing capabilities of the proposed setup (Figure 6a,b). As shown in Figure 5 and in [10], bias for the sensors is needed for the output to be linearized. Note that for all results, the real sensor sensitivity is shown (without amplification). As mentioned previously, the main challenge is low currents measurements, since higher currents can be easily detected with the setup from Figure 6 for example by integrating a copper bar on the PCB backside. In Figure 8, the response obtained with the setup by measuring a variable DC current between ±150 mA is shown. The sensitivity of the measured differential output is *S*_measured_ = 0.2319 mV/mA which shows a good correlation between the theoretical and experimental results. 

Compared with the results presented in [10], for the NVE AG003-01E sensor evaluation kit (which utilizes the same model of sensors), measured on a single trace with a similar width 0.254 mm, the obtained sensitivity is *S*_measured_ *=* 0.0179 mV/mA while for the differential system in [10] with a trace thickness of 4 mm, for the same 150 mA test, the obtained sensitivity *S*_D_ = 4 mm *=* 0.028 mV/mA which is approximately 8.3 times lower. Thus, in this test, with the multi-trace setup an increase in sensitivity of ~13 times compared to the sensor evaluation kit was obtained and ~8.3 times compared to the already optimized differential setup from [10]. Note that for easier comparison with the results that can be obtained with the analytical method, for each testing scenario, the sensitivity of the sensors is reported (the sensitivity of the entire setup depending on amplifier configuration).

**Figure 9:**
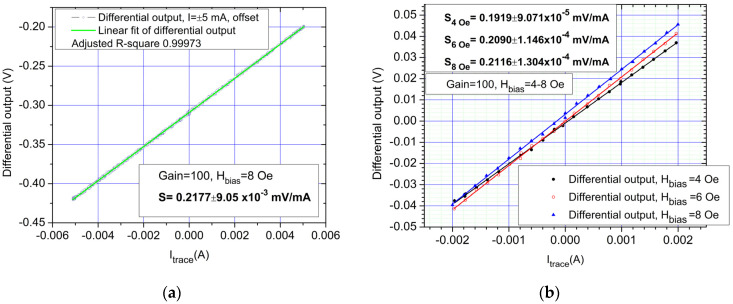
DC response of the system for low current values: (**a**) Differential output for a ±5 mA, DC current, *H_bias_* was set to 8 Oe; (**b**) Differential output of sensors polarized at 4,6,8 Oe, DC, ±2 mA (in this case, the adjusted R-squared for the fit function was: adjusted *R-square*_4Oe_ = 0.99961, adjusted *R-square*_6Oe_ = 0.9995, adjusted *R-square*_8Oe_ = 0.99943). Notice the linear characteristic of the output, although, for very low current values, some neliniarities can be present, but the overall linear tendency maintains.

Due to the significant gains in sensor sensitivity, lower current values can be detected accurately. In Figure 9a, the differential output obtained with the setup by measuring a variable DC current between ±5 mA. Figure 9b shows the differential response obtained from the setup measuring a variable DC current between ±2 mA with different biasing fields from 4–8 Oe. The results have shown the optimal sensitivity level to be around the 8 Oe bias level. Experiments with higher bias fields were performed, but the optimal sensitivity level was confirmed to be 8 Oe. Also, for a higher bias field and current values, especially with high amplifier gains, there could be a risk in saturating the voltage bandwidth for the amplifier or DAQ board. For measuring current values around 1 mA with good accuracy, extra precautions should be taken like electromagnetic shielding of the sensor setup and extra amplifications steps. The DC detection limit is 100 μA. 

For sensors that are perfectly matched and are subject to the same biasing field, the temperature drift of the offset can, theoretically go to zero. In [10] we measured the temperature drift of the offset to be ΔU_0_/ΔT≈ −7.9 × 10^−6^ V/°C which means about −2.59 × 10^−4^ A/°C in terms of measured current, for a temperature variation of 20 °C. Also, we can note that any temperature drifts in the operating range of the bias coils lead to no significant changes to the bias magnetic field as we estimate that the temperature of these components is no larger than 37 °C during our tests. Also, all measurements were performed on the setup at thermal equilibrium state, were no significant changes to the offset were observed. Furthermore, the low current values passing through the trace caused no significant heating effects on the PCB area in the GMR sensors vicinity.

**Figure 10:**
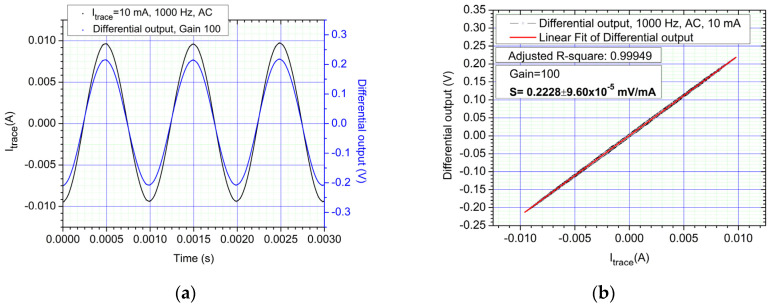
AC response of the system, 8 Oe biased sensors, 1000 Hz sine waveform at 10 mA: (**a**) Differential output and trace current time dependency; (**b**) Differential output of sensors. The sensitivity for the sensors in the case is *S* = 0.2228 mV/mA ±9.6 ×10^−5^. Notice the sensitivity level is similar to that of DC measurements.

In Figure 10a,b, the response of the system when measuring a 100 Hz, 10 mA sinewave current is shown. Similar sensitivities levels can be obtained for AC/DC currents but identical biasing fields must be applied for the two sensors. 

From Figure 10a,b, we can denote that the differential output maintains signal integrity (waveform of the trace current) with no distorsions. The detection limit for a 100 Hz sine wave is 100 μA (same as in DC) while for a 1 kHz sine wave is 300 μA (no significant increase in the detection limit was found at higher frequencies). We found this limitation to be due mainly to the signal processing chain and not due to the GMR sensors which have an operating frequency range from DC to 1 MHz [22].

**Figure 11:**
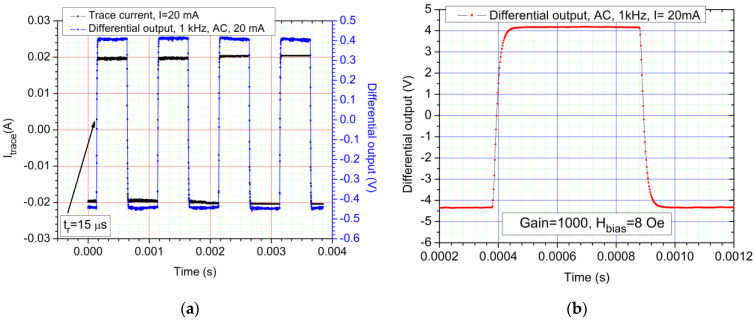
(**a**) AC response of the system, 8 Oe biased sensors, 1 kHz square waveform at 20 mA: a rise time and fall time of 15 µs was found in this case (measured between the 10–90% levels); The sensitivity for the sensors in this case is *S* = 0.2120 ± 7.186 × 10^−4^ mV/mA; (**b**) AC response of the system, 8 Oe biased sensors, AC, 1 kHz, 20 mA pulse.

**Figure 12:**
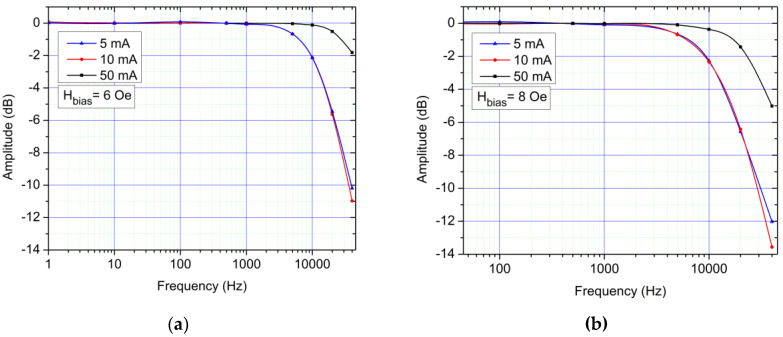
(**a**) AC frequency response (transfer function) of the system, 6 Oe biased sensors for different measured currents; (**b**) AC frequency response (transfer function) of the system, 8 Oe biased sensors for different measured currents.

The AC response to a square wave, 1 kHz, 20 mA current is detailed in Figure 11a. Multiple tests (with different biasing levels and frequencies) have determined a rise time and fall time of approximately 15 µs. Figure 11b shows the response of the system under a short 20 mA square pulse. Figure 12a,b show that even though, at lower frequencies, 8 Oe was the optimal choice in terms of biasing, at higher frequencies, 6 Oe is optimal in the present setup (from the 4–8 Oe bias fields range). From this analysis, we determined that, as expected, the rise and fall times as well as the frequency characteristics of the system this is mainly limited by the electronics (especially the instrumentation amplifiers) as the GMR sensors have a maximum frequency response limit of 1 MHz [22].

**Figure 13:**
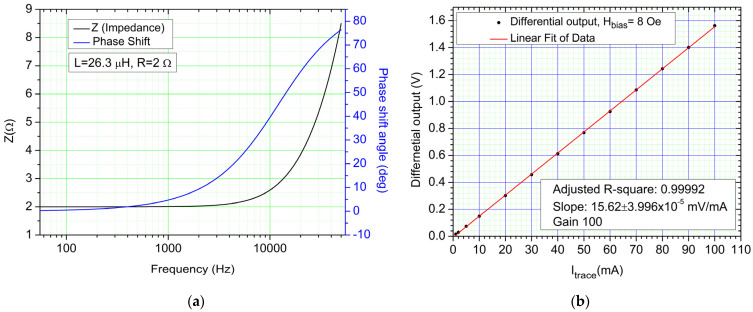
(**a**) Impedance-frequency characteristic and current-voltage phase shift angle frequency dependency; (**b**) AC calibration curve in the 0–100 mA range for a 100 Hz sinewave. Note that the minimum trace current represented on the calibration curve is 1 mA.

Since the planar coil has an inductive component under AC (inductance of 26.3 µH), impedance can play a role in the response of the system. The impedance-frequency (Figure 13a) characteristic can create a phase-shift between the current and voltage waveforms. The impedance of the planar coil is equal to the resistance up to around 4000 Hz (*Z* = *R* = 2 Ω).

Figure 13b shows the AC calibration curve for the device within the 0–100 mA range when measuring a 100 Hz sinewave. We used the adjusted R-squared term to show how well data is aligned over the fitting line. The adjusted *R-square* is 0.99992. The sensitivity of the entire setup in *S*, in the 0–100 mA range is 15.62 mV/mA. Note that there is a very good correlation between the measured current and the response of the system.

### 3.2. Experimental Results for the Magnetic Nanoparticles Detection Setup

**Figure 14:**
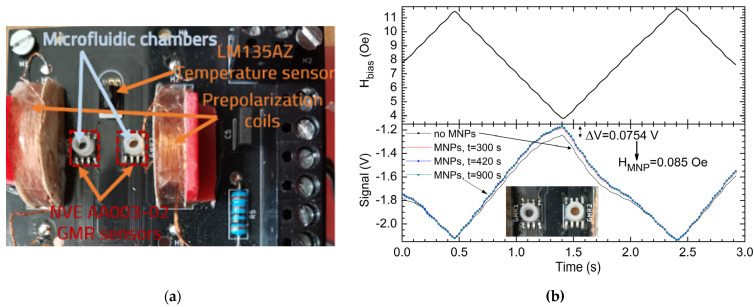
(**a**) Measurement system adapted for the detection of MNP (**b**) Waveform of the applied biasing field, *H_bias_* and output signals from setup: with standardized distilled water probe and MNP aqueous solution over the sensor’s surface after different elapsed times. The greatest field contribution of the MNPs was found at the *H_bias_* = 4 Oe level where a ΔU = 0.0754 V signal variation was found compared with the case with no MNPs. The total gain of the signal was *G* = 100.

Even though the developed system is not designed for magnetic nanoparticles (MNPs) detection (due to the design of the GMR sensors), it can still be used for proof-of-concept purposes. Figure 14a shows the measurement setup for the MNPs detection setup, while Figure 14b shows the waveform of the applied biasing field, *H_bias_* and output signals from setup with standardized distilled water probe and MNP aqueous solution. Thus, for this purpose, on the setup (Figure 6a), two cylindrical chambers with the interior diameter of 2.5 mm and 2 mm height were attached on top of the sensors—a reference chamber on S_1_, which will contain water, and in the other chamber (on S_2_) an aqueous solution with PEG6000 functionalized maghemite MNPs will be pipetted (note the inset in Figure 14b). The MNPs in the aqueous solution functionalized with PEG6000 have an average magnetic diameter, d_magn_, of 11.48 nm which has been determined in a previous study [27]. 

In both chambers, the same amount of liquid was pipetted: 2.5 μL distilled water on sensor 1, and the previously described aqueous MNPs solution on sensor 2. In this way, the thermal balance for the two sensors can be ensured. The system was biased at 8 Oe, then the current through the biasing coils was varied in steps at different values in the 26–84 mA interval similar to the characteristic in Figure 9b. This generated a variable magnetic field in the 3.61–11.76 Oe region, centered on 8 Oe. The graph in Figure 14b, marks different values for the biasing field. Note that this still represent a DC test, with a variable biasing level. Due to the differential measurement setup, the differential output voltage is an expression of the magnetic field generated by the magnetic nanoparticles situated on sensor 2 (Figure 14b). Given the return field lines, the effective magnetic field from sensor 2 will decrease. Figure 14b shows the detection characteristic obtained on different time intervals after pipetting the MNPs solution on sensor 2. 

In this way, given the calibration process described above, the magnetic field generated by the MNPs on sensor 2 was estimated (Figure 14b). For “large” fields like 12 Oe, the contribution of the field produced by the MNP is covered by the biasing field. By analyzing Figure 14b, we can notice that the field contribution of the MNPs is greatest at a 4 Oe biasing field. Thus, for *H*_bias_ = 4 Oe, the MNPs generate a magnetic field of approximately *H* = 0.085 Oe. Using data from the magnetization curves [27] of the PEG6000 functionalized MNPs we estimated that our system determined a magnetic moment of about 0.29 × 10^−4^ emu for a signal variation of 0.0754 V. This magnetic moment corresponds to a mass of about 33.39 μg of powder composed from maghemite functionalised with PEG 6000. This means about 2.40 μg of pure maghemite cores. The estimation was done by comparing the magnetization curves for pure maghemite powder [28] and functionalised maghemite with PEG 6000 [27,28]. 

By varying the biasing field, an unequivocal detection of the presence of MNPs is obtained. The tests highlight the MNPs sedimentation process on the surface of the sensor through the amplitude of the measured signal. In [29], a detection system utilizing the same type of sensor is shown but, in that case, the MNPs solution is placed on a cylinder which rotates in the sensor vicinity (the system detects the magnetic field variations as the probe passes the sensor). Our proposed solution does not require any moving parts and allows great flexibility in the MNPs detection regime as well as possibility for autocalibration by means of the planar coil which generates a local magnetic field, similar to that generated by the MNPs.

### 3.3. State of the Art Comparison

Although the developed setup is for demonstration purposes and is not intended to be compared with commercial sensor solutions, a summarizing state-of-the-art performance comparison with similar magnetoresistive sensor technologies is given in Table 1. 

It should be mentioned that performance characteristics are dependent on the setup, electronics and configuration, and also are defined differently depending on the manufacturer. Thus, direct comparison can prove difficult and some parameters cannot be determined precisely. Table 2 shows the main advantages and disadvantages of the implemented setup. From Table 1 and Table 2, we can note the performance advantage of the implemented setup, especially in terms of sensitivity, detection limit and power consumption. Also, the developed setup is much more flexible in terms of applications as the sensors can be precisely calibrated using the biasing coils and it can also be used for MNPs measurements.

In terms of drawbacks, the biasing coils consume the majority of power in our setup, but this effect can be mitigated by replacing them with a permanent magnet in applications that do not require a variable biasing level. Also, the proposed system is a hybrid setup with moderate integration level meaning that future efforts can focus on compactness and versatility in component choice and placement. We do not consider the specified measurement range a disadvantage since can be easily extended for higher currents by integrating a thicker copper trace or bar (Table 1, footnote 1). 

## 4. Conclusions

A very high sensitivity non-contacting current measurement setup based on a custom PCB with GMR sensors, which is optimized for low field applications was implemented. The system is designed to measure currents between 2–300 mA but the operational range can be extended, for example by integrating a copper bar on the PCB backside (Figure 6a). The setup has a sensitivity between 15.62 to 23.19 mV/mA, for biasing fields between 4 to 8 Oe with a detection limit of 100 μA in DC and 100 to 300 μA in AC from 10 Hz to 50 kHz. The reported sensor sensitivity is about 13 times higher than a single similarly biased GMR sensor and around 7 to 8.5 times increase in sensitivity compared to the optimized differential setup that we showed in [10]. A biasing field applied by two circular coils in a quasi-Helmholtz like configuration were used to linearize the system response and allow different modes of operation (different biasing fields, variable biasing fields). This approach was taken to increase the versatility of the system as a testing environment, but for a practical application, a permanent magnet has many advantages such as no extra power consumption or generated heat. The novelty of our approach consists in using a multi-trace setup that essentially constitutes a planar coil which will increase the useful magnetic field in the sensor area. An analytical method was implemented (Figure 2a, Equations (2)–(7)) to estimate this increase. Also, the sensors operate in a similar double differential setup to the one we reported in [10]. Together, this has greatly improved the operational range of the sensor for low current values. This approach was not seen in other works [9,17,18,19], or in commercial sensor solution based on AMR [8,30,33], Hall [31], or microfluxgate [4,6,34]. The obtained performance makes this setup suitable to be adapted and implemented in various current measurement applications for high precision electronics, smart grid applications and automotive industry.

The results were obtained without using electromagnetic shielding and for AC measurements, a basic integrated capacitor filtering system was used. It was determined that the AC frequency characteristics are mostly limited by the electronics (amplifier system). The impedance of the planar coil has a significant influence only after frequencies above 4000 Hz. The system exhibits certain qualities such as: high sensitivity (similar sensitivity levels for DC and AC currents), galvanic isolation, thermal stability (within the operating limits), preservation of signal integrity from the input current (Figure 10 and Figure 11). The power rating of the system is very low since the sensors consume only about 6.4 mW (3.2 mW each), the biasing coil system consumes around 251.7 mW for an 8 Oe bias field, each INA118 has a quiescent current of only 350 µA and the LabJack EI1040 is very low power. The most energy consuming element in the current setup is the biasing coil system which can be easily replaced with a small permanent magnet (as shown in [10]) for each sensor in case the application does not require an AC biasing field. Consequently, the system can be described as very low power. 

Furthermore, this field and sensitivity is sufficient for the detection of MNPs with the GMR sensor [35], thus, preliminary testing using the setup for an aqueous solution of PEG6000 functionalized maghemite magnetic nanoparticles was performed. With this setup, the magnetic field of the nanoparticles of about 0.085 Oe was able to be detected reliably (Figure 13b) on a standardized sample. We estimate that with a sensor design optimized for nanoparticles detection the performance can be improved even more. The current through the conductive band can be used to produce an AC excitation field for detection of the MNPs. To ensure a smaller distance between MNPs and GMR sensors, a flip-chip package type can be used in this development, as in [36]. This approach will reduce the distance between the MNPs and the sensor, which will improve sensitivity and avoid utilizing complex measurement setups like in [29], where the MNP solution is placed in a container on a rotating cylinder in the sensor’s vicinity. In that case the system detects the magnetic field variations caused by the probe passing by the sensor. Our proposed solution does not require any moving parts and allows great flexibility in choosing the MNPs detection regime as well as autocalibration function through the planar coil which generates a local field similar to the one generated by the MNPs.

Finally, the theoretical (analytical method) and operational basis (practical implementation) for developing a multi-trace GMR-based high sensitivity current sensor PCB has been established. From those we can note: appropriate biasing of the sensors, adequate spacing of components to avoid parasitic magnetic fields, multi-trace and differential or double differential design, appropriate amplification, filtering and quality data acquisition. In terms of future developments, an optimized, chip sensor design that will integrate many of the advancements from the setup will developed which can increase the low field capabilities even further.

## Figures and Tables

**Table 1:** Summarizing state-of the art performance comparison for magnetoresistive current sensor technologies.

Parameter	This Work	[10]	[9]	MCA1101-xx-5 Series [30]	TMCS1100A Series [31]	ACS70331 Series [32]	[14]
Sensor technology	GMR	GMR	GMR	AMR	Hall	GMR	PHR (planar Hall resistance)
Sensor setup sensitivity	0.1562 to 0.2319 mV/mA	0.0272 to 0.0307 mV/mA	0.03 to 0.04 V/A ^3^	35 mV/A to 350 mV/A	50 to 400 mV/A	200 to 800 mV/A	1.2 mA/LSB (12 bit)
Measurement range	DC: ±2 mA to ±300 mA ^1^ AC: ±2 mA to ±300 mA ^1^	DC: ±75 mA to ±4 A AC: ±150 mA to ±4 A	±45 A	±5 to ±50 A	±5.75 A to ±46 A	0–2.5 A to ±5 A	±1.2 A
Detection limit	DC: 100 µA AC: 100 to 300 µA	4 mA	N/A	10 mA	N/A	5 mA	5 mA
Power consumption	Setup: ~258 mW ^2^ Sensors: 6.4 mW ^2^	~6.4 mW	1.6 to 3.2 W	~32.5 to 35 mW	33 mW (no V_out_ load); 640 mW ^4^	14.9 mW	13 mW
Calibration	Yes, biasing coil system	Yes, adjustable permanent magnet	Yes, biasing coil system	N/A	N/A	Yes, Analog-to-digital converter	Yes, Analog-to-digital converter

^1^ Note that the measurement range can be easily extended by integrating a thicker copper trace or bar (e.g. on the PCB backside or above the sensor) to support higher currents. The focus of this work was to increase accuracy in the low currents range. ^2^ The power consumption of the setup can be reduced to ~6.4 mW by replacing the biasing system with a permanent magnet. Note that the power consumption of the biasing coils is ~251.7 mW for a 57.55 mA current (corresponding to an 8 Oe bias field) passing through the coils while the GMR sensors dissipate around 6.4 mW. ^3^ Not directly specified in the article but can be deducted from experimental results. ^4^ Maximum power dissipated when measuring a current of 16 A.

**Table 2:** Main advantages and disadvantages of the implemented setup.

Advantages	Disadvantages
High sensor sensitivity: 0.1562 to 0.2319 mV/mA	Limited measurement range (2–300 mA) ^1^
Low detection limit: DC: 100 µA AC: 100 to 300 µA	Coil biasing system consumes extra power ^2^
Precision biasing with coils	Hybrid setup ^3^
Precision DC/AC current sensing	Moderate components integration level
Moderately low power consumption (note Table 1)	-
Possibility for MNPs measurements	-
Low temperature drift of the offset: −2.59 × 10^−4^ A/°C	-

^1^ The measurement range can be easily extended (note Table 1, footnote 1). ^2^ Biasing coils can be replaced by a permanent magnet for applications that do not require a variable biasing field. ^3^ The sensors setup is separate from the amplifier and data acquisition setup.

## Appendix A

In this appendix section, an example calculus for the analytical method is shown. The parameters involved in the calculation are shown in Table A1 (note Figure 2a).

**Table A1:** Parameters for an example calculation of the analytical method.

Parameter	*D* [mm]	*T*_d_ [mm]	I [A]	µ_0_ [H/m]	h [mm]	*D_n_*_1_ [mm]	*D_n_*_2_ [mm]
Value	0.22	0.19	0.5	4π × 10^−7^	0.8	Equation (A1)	Equation (A2)

Parameters *D_n_*_1_ and *D_n_*_2_ are detailed in Equations (A1) and (A2).

Parameters *D_n_*_1_, *D_n_*_2_ can be computed in function of the number of traces, *n* (Figure 2a), with:

(A1) $$D_{n1}=\frac{D}{2}+\left(n-1\right)D+nT_{d}\;,$$

(A2) $$D_{n2}=\frac{D}{2}+nD+nT_{d}\;.$$

Based on Equations (A1) and (A2), the individual *D*_n1_ and *D*_n2_ values can be calculated. Furthermore, we can substitute them in Equation (4) for calculating the magnetic field produced by each trace, whilst doubling the field value for the field produced by a similarly distanced traced from the sensor. The calculation for the individual trace components, based on the parameters in Table A1 is:

(A3) $$B_{0}=\frac{\mu_{0}I}{\pi D\cdot 10^{-3}}\cdot \left[arctan\left(\frac{D}{2h}\right)\right]=1.2422\cdot 10^{-4}\;\left[T\right],$$

(A4) $$2\cdot B_{1}=\frac{\mu_{0}I}{\pi D\cdot 10^{-3}}\cdot \left[arctan\left(\frac{D_{12}}{h}\right)-arctan\left(\frac{D_{11}}{h}\right)\right]=1.9782\cdot 10^{-4}\;\left[T\right],$$

(A5) $$2\cdot B_{2}=\frac{\mu_{0}I}{\pi D\cdot 10^{-3}}\cdot \left[arctan\left(\frac{D_{22}}{h}\right)-arctan\left(\frac{D_{21}}{h}\right)\right]=1.223\cdot 10^{-4}\;\left[T\right],$$

(A6) $$2\cdot B_{3}=\frac{\mu_{0}I}{\pi D\cdot 10^{-3}}\cdot \left[arctan\left(\frac{D_{32}}{h}\right)-arctan\left(\frac{D_{31}}{h}\right)\right]=7.457\cdot 10^{-5}\;\left[T\right],$$

(A7) $$2\cdot B_{4}=\frac{\mu_{0}I}{\pi D\cdot 10^{-3}}\cdot \left[arctan\left(\frac{D_{42}}{h}\right)-arctan\left(\frac{D_{41}}{h}\right)\right]=4.81\cdot 10^{-5}\;\left[T\right],$$

(A8) $$2\cdot B_{5}=\frac{\mu_{0}I}{\pi D\cdot 10^{-3}}\cdot \left[arctan\left(\frac{D_{52}}{h}\right)-arctan\left(\frac{D_{51}}{h}\right)\right]=3.31\cdot 10^{-5}\;\left[T\right].$$

With the results from Equations (A3)–(A8), we can compute the total field corresponding to a particular multi-trace structure (note that *B*_0_ is the result for a single trace). The result is shown in Equations (A9)–(A13):

(A9) $$3\;\mathrm{spires}:\;B_{total}=B_{0}+2B_{1}=3.2204\cdot 10^{-4}\;\left[T\right]$$

(A10) $$5\;\mathrm{spires}:\;B_{total}=B_{0}+2B_{1}+2B_{2}=4.4434\cdot 10^{-4}\;\left[T\right]$$

(A11) $$7\;\mathrm{spires}:\;B_{total}=B_{0}+2B_{1}+2B_{2}+2B_{3}=5.1891\cdot 10^{-4}\;\left[T\right]$$

(A12) $$9\;\mathrm{spires}:\;B_{total}=B_{0}+2B_{1}+2B_{2}+2B_{3}+2B_{4}=5.6701\cdot 10^{-4}\;\left[T\right]$$

(A13) $$11\;\mathrm{spires}:\;B_{total}=B_{0}+2B_{1}+2B_{2}+2B_{3}+2B_{4}+2B_{5}=6.0011\cdot 10^{-4}\;\left[T\right]$$
